# An Extensive Gap Junction Neural Network Modulates *Caenorhabditis elegans* Aversive Behavior

**DOI:** 10.3390/genes16030260

**Published:** 2025-02-23

**Authors:** Savannah E. Sojka, Meredith J. Ezak, Emily A. Polk, Andrew P. Bischer, Katherine E. Neyland, Andrew P. Wojtovich, Denise M. Ferkey

**Affiliations:** 1 Ferkey Laboratory, Department of Biological Sciences, University at Buffalo, The State University of New York, Buffalo, NY 14260, USA; 2 Wojtovich Laboratory, Department of Anesthesiology and Perioperative Medicine, University of Rochester Medical Center, Rochester, NY 14642, USA

**Keywords:** innexin, gap junction, cGMP, behavior, chemosensation, nociception, avoidance, *C. elegans*

## Abstract

Background/Objectives: *Caenorhabditis elegans* rely on sensory perception of environmental cues for survival in their native soil and compost habitats. These cues provide information about nutrient availability, mating partners, or predatory and hazardous beacons. In *C. elegans*, the two bilaterally-symmetric head sensory neurons termed ASH are the main detectors of aversive nociceptive signals. Through their downstream connections in the nervous system, ASH activation causes the animal to initiate backward locomotion to escape and avoid the harmful stimulus. Modulation of avoidance behavior allows for situation-appropriate sensitivity and response to stimuli. We previously reported a role for gap junctions in the transport of regulatory cGMP to the ASHs where it functions to dampen avoidance responses. Methods: Here, we used genetic mutants and a combination of cell-selective rescue and knockdown experiments to identify gap junction proteins (innexins) involved in modulating ASH-mediated nociceptive behavioral responses. Results: We have characterized six additional *C. elegans* innexins that have overlapping and distinct roles within this regulatory network: INX-7, INX-15, INX-16, INX-17, UNC-7, and UNC-9. Conclusions: This work expands our understanding of the extent to which ASH sensitivity can be tuned in a non-cell-autonomous manner.

## 1. Introduction

Soluble and volatile chemical cues can signal favorable environmental conditions to an organism, such as the presence of food or mates, as well as potential dangers to be avoided. Response to these cues begins with detection by specialized sensory cells in the periphery. However, we are only beginning to understand how the signals are relayed, integrated and tuned to elicit appropriate behavioral responses as the presence of both chemical and electrical synapses between neurons results in richly complex circuits through which information can pass.

Chemical synapses allow for both short-term and long-term signaling events through the vesicular release of neurotransmitters into synaptic clefts between cells. In contrast, gap junctions are composed of channels that link across neighboring cell membranes to allow for electrical coupling and direct passage of small cytoplasmic molecules, such as ions, metabolites, mRNAs, and cyclic nucleotides [1,2,3,4,5,6,7,8,9]. In vertebrate systems, six connexin proteins come together in the cell membrane to form one hemichannel with a central pore. Although hemichannels can act independently to shuttle biomolecules in or out of a single cell [10,11], two cells become functionally connected via a gap junction when a hemichannel from one cell associates with a hemichannel from another cell. Gap junctions can exist in a variety of configurations, including homomeric/homotypic (made up of a single protein subunit type in both cells), homomeric/heterotypic (consisting of two different homomeric hemichannels in the two cells), or heteromeric/heterotypic (made up of a mixture of subunit types in both cells) [12,13,14,15]. The *C. elegans* genome encodes 25 innexins (invertebrate analogues of the connexins) [16], highlighting the significant role they likely play in transducing or regulating neuronal signaling in this model system [17].

The primary nociceptors in *C. elegans* are the two bilaterally-symmetric head sensory neurons termed ASH. These neurons exhibit wide ranging functionality as they aid in the detection of soluble, volatile and physical aversive stimuli, including: tastants, odorants, pH changes, mechanical stimulation, and osmotic pressure [18,19,20,21,22,23,24,25,26,27,28,29,30,31,32,33]. In response to any of these stimuli, the nematode will initiate backward locomotion to escape the stimulus source. Bitter tastants and the odorant 1-octanol are believed to activate G protein-coupled signaling cascades in ASH [34], although the identity of the *C. elegans* receptors for these stimuli remains unknown. Although nociception is a protective behavioral response, an animal’s sensitivity to noxious stimuli is tuned to its internal state to allow for context-appropriate behavioral responses. For example, *C. elegans* are more responsive to aversive cues when they are well fed [35,36,37,38,39,40]. Upon food deprivation, aversive responses are dampened, which may increase the chances that animals will enter new environments, even if not favorable, where they could encounter a food source.

Our previous work revealed a role for cGMP and an innexin-based neural network in modulating nociceptive avoidance responses and suggested that upon food deprivation, the ODR-1 guanylyl cyclase produces cGMP in AWB, AWC and ASI, which then moves through gap junctions to ASH [41]. Within ASH, cGMP binds to the cGMP-dependent protein kinase (PKG) EGL-4, likely stimulating it to phosphorylate and activate RGS-2 and RGS-3, which then dampen G protein-coupled signaling at the level of Gα protein subunits downstream of the activated receptor. In this way, cGMP produced non-cell-autonomously serves to dampen ASH signaling and behavioral sensitivity to aversive stimuli [40,41,42,43]. Accordingly, loss-of-function mutations in guanylyl cyclases that produce this pool of cGMP (*odr-1*, and also likely *gcy-27*, *gcy-33*, *gcy-34*), innexins that facilitate its transport (*inx-4*, *inx-18*, *inx-19*, *inx-20*), or the regulatory components (*egl-4*, *rgs-2*, or *rgs-3)* all result in elevated behavioral responses to ASH-detected chemical stimuli, which we refer to as hypersensitivity [40,41,42,43].

Although many sensory neurons (AWB, AWC, ASI, ADF, AFD, ADL, ASJ and ASK) and interneurons (AIA and RMG) function in this modulatory network, the sites of action for only four innexins within it have been determined via loss-of-function mutants and cell-selective rescue experiments (Supplementary Figure S1). INX-4 functions in ASH, likely allowing the final inflow of cGMP into this neuron [40], INX-18 functions in ASK (which forms gap junctions with ASH), INX-19 functions in both ASK (which forms gap junctions with ASH) and ASH, and INX-20 functions in ADL and AFD [42,43]. ADL forms gap junctions with RMG and ASK, which both form connections with ASH, while AFD is directly connected to ASH. RNAi data also suggested a role for INX-7 in RMG [43].

Extending previous work [40,42,43], here we complete the survey of all 25 innexin genes, and have identified modulatory roles for INX-7, INX-15, INX-16, INX-17, UNC-7, and UNC-9. Animals lacking any of these innexins are hypersensitive to a dilute concentration of the bitter tastant quinine, and a combination of cell-selective RNAi and rescue experiments revealed the site(s) of function for each.

## 2. Materials and Methods:

### 2.1. C. elegans Culture

Strains were maintained under standard conditions on NGM agar plates seeded with OP50 *Escherichia coli* bacteria [44]. For a list of strains used in this study, see Supplementary Information.

### 2.2. Behavioral Assays

Well-fed young adult *C. elegans* animals grown at 20 °C were used for analysis, and all behavioral assays were performed on at least three separate days, in parallel with controls. Response to quinine was scored as the percentage of animals on a plate that initiated backward locomotion within 4 s of encountering a drop of the stimulus placed on the agar plate in front of a forward moving animal [22,23]. Each plate counted as one replicate, performed at least in duplicate each day, over at least 3 days. For rescue and RNAi knockdown experiments, multiple transgenic lines were pooled for analysis. Our experiments used a “wet drop” that animals entered instead of a “dry drop”, and each animal was tested only once [40,41,43,45,46]. Quinine was dissolved in M13 buffer, pH 7.4 [47]. Animals were tested 30 min after transfer to NGM plates lacking bacteria (“off food”). All data are presented as ± standard error of the mean (SEM). One-way ANOVA with Tukey’s Honestly Significant Difference (HSD) was used for statistical analyses. In all figures, * denotes *p* < 0.05, ** denotes *p* < 0.01, *** denotes *p* < 0.001 and **** denotes *p* < 0.0001. ns denotes *p* ≥ 0.05.

### 2.3. Plasmid Construction

For a list of plasmids generated for this study see Supplementary Information.

### 2.4. Transgenic Strain Generation

All cell-specific RNAi was performed as previously described [48]. Injection of each fusion product was performed at 50 ng/uL with the inclusion of 50 ng/uL of pJM *elt-2::gfp* plasmid used as a visible co-injection marker [49]. Germline transformations were performed as previously described [50]. All cDNA rescue constructs were injected at a 50 ng/uL concentration, with the exception of locomotor-rescue plasmids, *glr-1p::unc-7* and *unc-4p::unc-9*, which were injected at 75 ng/uL and 15 ng/uL, respectively.

### 2.5. Supplemental Information

For information on *C. elegans* strains (including transgenic strains) and plasmids, see Supplementary Information.

## 3. Results

### 3.1. The INX-7, INX-15, INX-16 and INX-17 Gap Junction Components Function in Distinct Subsets of Neurons to Modulate Quinine Sensitivity

**Figure 1:**
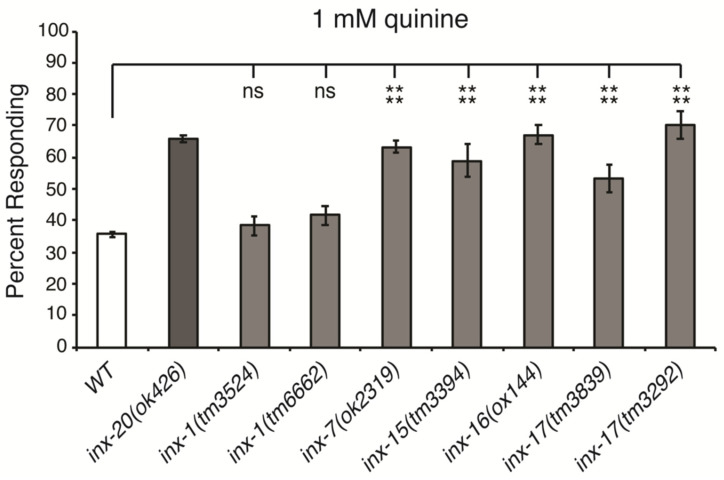
INX-7, INX-15, INX-16 and INX-17 regulate quinine sensitivity. Loss-of-function mutations in the innexin gap junction genes *inx-7*, *inx-15*, *inx-16* and *inx-17* resulted in behavioral hypersensitivity to 1 mM quinine (*p* < 0.0001 for each when compared to wild-type animals). Loss of *inx-1* function did not affect quinine sensitivity (*p* > 0.6 for both alleles when compared to wild-type animals). The percentage of animals responding is shown. Error bars represent the standard error of the mean (SEM). WT = the N2 wild-type strain. **** denotes *p* < 0.0001. ns denotes *p* ≥ 0.05 (not significant).

To determine whether any of the remaining untested innexins might also modulate quinine sensitivity, we first tested animals with loss-of-function alleles for *inx-1*, *inx-7*, *inx-15*, *inx-16* or *inx-17* for response to dilute (1 mM) quinine. Each of these homozygous strains was viable and while *inx-16(lof)* and *inx-17(lof)* had somewhat delayed growth, none displayed morphological or movement defects that would impede behavioral assays. Two different alleles of *inx-1* were available, and both responded similarly to wild-type animals (Figure 1). In contrast, *inx-7(lof)*, *inx-15(lof)*, *inx-16(lof)* and *inx-17(lof)* animals were all hypersensitive in their response to dilute quinine (Figure 1).

**Figure 2:**
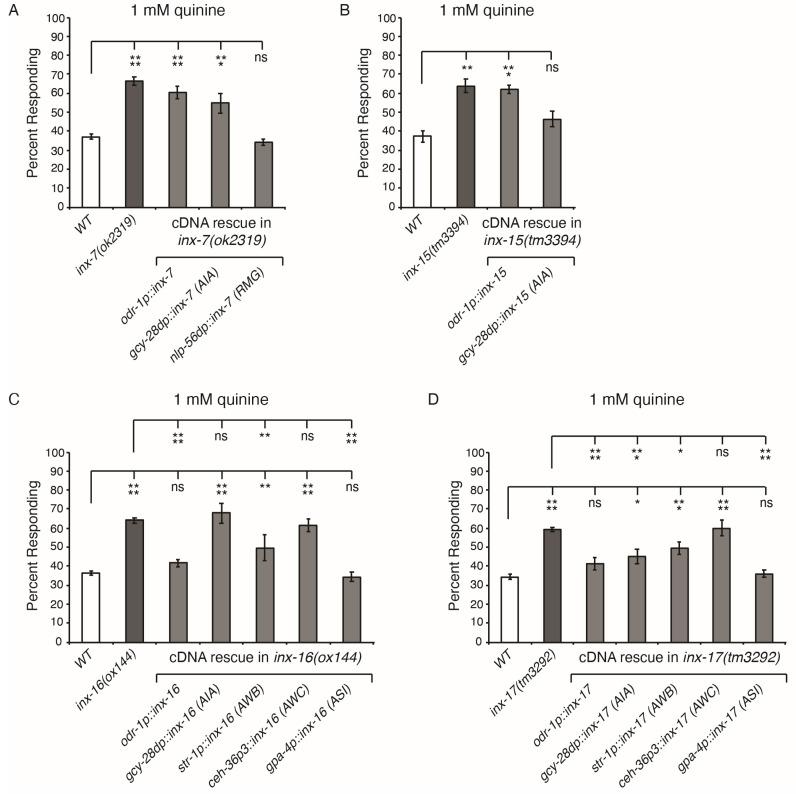
INX-7, INX-15, INX-16 and INX-17 sites of action. Cell-selective promoters were used to rescue innexin function in the respective loss-of-function backgrounds. (**A**) Expression of *inx-7* cDNA in RMG using the *nlp-56* promoter [54,55] fully rescued quinine hypersensitivity (*p* > 0.9 when compared to wild-type animals). (**B**) Expression of *inx-15* cDNA in AIA using the *gcy-28d* promoter [53] fully rescued quinine hypersensitivity (*p* > 0.3 when compared to wild-type animals). (**C**) Expression of *inx-16* cDNA using the *odr-1* promoter (AWB, AWC, ASI, ASJ and ASK) [51] restored the response to wild-type levels (*p* > 0.8). Expression in AWB using the *str-1* promoter [56] partially rescued hypersensitivity [*p* < 0.01 when compared to either wild-type or *inx-16(lof)* animals], while expression in ASI using the *gpa-4* promoter [58] fully rescued hypersensitivity (*p* > 0.9 when compared to wild-type animals). (**D**) The *inx-17(tm3292)* allele was chosen for the rescue experiments due to its stronger hypersensitive phenotype as seen in Figure 1. Expression of *inx-17* cDNA (encoding the a isoform) using the *odr-1* promoter restored the response to wild-type levels (*p* > 0.1). Expression in AIA or AWB partially rescued hypersensitivity [*p* < 0.05 when compared to either wild-type or *inx-17(lof)* animals]. Expression in ASI restored the response to wild-type levels (*p* > 0.9). The combined data of ≥3 independent lines and n ≥ 90 transgenic animals are shown for each rescue. In all graphs the percentage of animals responding is shown. Error bars represent the standard error of the mean (SEM). WT = the N2 wild-type strain. * denotes *p* < 0.05, ** denotes *p* < 0.01, *** denotes *p* < 0.001 and **** denotes *p* < 0.0001. ns denotes *p* ≥ 0.05 (not significant).

To identify the site of action for each of these four innexins in modulating quinine sensitivity, cell-selective promoters were used to restore function in the respective loss-of-function genetic backgrounds. Expression of *inx-7* cDNA in *inx-7(lof)* animals using the *odr-1* promoter (AWB, AWC, ASI, ASJ and ASK) [51] did not rescue the hypersensitive response to dilute (1 mM) quinine (Figure 2A). Expression in the AIA interneurons, which lie between each of the *odr-1-*expressing neurons and ASH [52] (Supplementary Figure S1 and Wormwiring.org), using the *gcy-28d* promoter [53], also did not rescue hypersensitivity Figure 2A). However, consistent with our previous RNAi analysis [43], expression of *inx-7* in the RMG neurons of *inx-7(lof)* animals, using the *nlp-56* promoter [54,55], restored the response to wild-type levels (Figure 2A).

Using the *odr-1* promoter to drive expression of *inx-15* cDNA in *inx-15(lof)* animals did not rescue quinine hypersensitivity, while expression in AIA (*gcy-28dp*) did (Figure 2B). In contrast, expression of either *odr-1p::inx-16* or *odr-1p::inx-17* (cDNAs) in the corresponding loss-of-function background rescued quinine hypersensitivity and restored the response to wild-type levels (Figure 2C and Figure 2D, respectively). Of the neurons covered by the *odr-1* promoter, AWB, AWC and ASI were previously shown to modulate quinine avoidance [40]. When testing *inx-16* rescue in each of these neurons individually, AWB expression using the *str-1* promoter [56] partially rescued quinine hypersensitivity, AWC expression using the *ceh-36p3* promoter [57] had no effect, and ASI expression with the *gpa-4* promoter [58] fully restored the response to wild-type levels (Figure 2C). For *inx-17* rescue, AWB expression partially rescued hypersensitivity, AWC expression had no effect, and ASI expression fully restored the response to wild-type levels (Figure 2D). While expression of *inx-16* cDNA in the AIA (*gcy-28d* promoter) neurons of *inx-16(lof)* animals had no effect, expression of *inx-17* in AIA of *inx-17(lof)* animals significantly dampened hypersensitivity (Figure 2C,D). Combined, these data reveal overlapping yet distinct sites of action for four innexins not previously known to play a role in regulating aversive behavior.

### 3.2. RNAi Reveals a Role for UNC-7 and UNC-9 in Modulating Quinine Sensitivity

**Figure 3:**
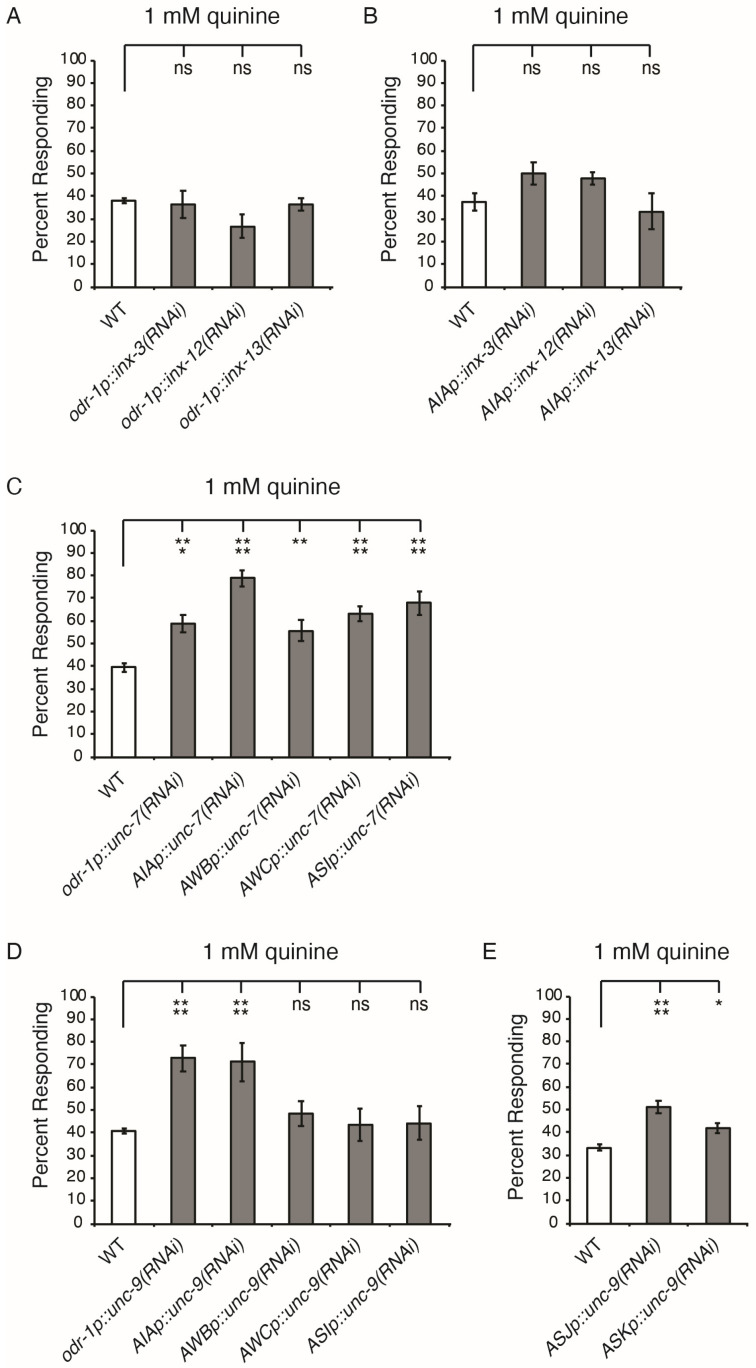
Cell-selective knockdown of *unc-7* or *unc-9* results in quinine hypersensitivity. RNAi knockdown of *inx-3*, *inx-12*, or *inx-13* using either (**A**) the *odr-1* promoter (AWB, AWC, ASI, ASJ and ASK) [51] or (**B**) the *gcy-28d* AIA promoter [53] did not result in quinine hypersensitivity (*p* > 0.15 for each when compared to wild-type animals). (**C**) RNAi knockdown of *unc-7* using either the *odr-1* promoter or the *gcy-28d* AIA promoter led to quinine hypersensitivity (*p* < 0.001). Cell-specific knockdown using the AWB *str-1* promoter [56], the AWC *ceh-36p3* promoter [57], or the ASI *gpa-4* promoter [58] also resulted in hypersensitivity (*p* < 0.01). (**D**) RNAi knockdown of *unc-9* using either the *odr-1* promoter or the *gcy-28d* AIA promoter led to quinine hypersensitivity (*p* < 0.0001), while knockdown in AWB, AWC or ASI did not (*p* > 0.7 for each when compared to wild-type). (**E**) RNAi knockdown of *unc-9* using either the ASJ *trx-1* promoter [61] or the ASK *srbc-66* promoter [62] led to partial hypersensitivity (*p* < 0.05 for each). The combined data of ≥3 independent lines and n ≥ 90 transgenic animals are shown. In all graphs, the percentage of animals responding is shown. Error bars represent the standard error of the mean (SEM). * denotes *p* < 0.05, ** denotes *p* < 0.01, *** denotes *p* < 0.001 and **** denotes *p* < 0.0001. ns denotes *p* ≥ 0.05 (not significant).

Of the 25 innexins expressed in *C. elegans*, three candidates lacked viable loss-of-function alleles (*inx-3*, *inx-12* and *inx-13*), while mutation of *unc-7 or unc-9* resulted in severe locomotive defects [44,59,60]. To examine the contribution of these innexins to avoidance modulation, we conducted a series of RNA-interference (RNAi) experiments (Figure 3), which did not affect the development or locomotion of the transgenic animals. Using neither the *odr-1* promoter (AWB, AWC, ASI, ASJ, ASK) nor the *gcy-28d* promoter (AIA) to knock down *inx-3, inx-12* or *inx-13* resulted in a hypersensitive phenotype (Figure 3A,B). However, when we used these same promoters to knock down either *unc-7* or *unc-9*, quinine hypersensitivity was observed (Figure 3C,D). Cell-specific knockdown of *unc-7* in AWB using the *str-1* promoter [56], AWC using the *ceh-36p3* promoter [57], or ASI using the *gpa-4* promoter [58], all led to hypersensitivity (Figure 3C). In contrast, single-cell knockdown of *unc-9* in AWB, AWC or ASI did not result in elevated response to quinine (Figure 3D). However, two additional neurons (ASJ and ASK) were covered by the *odr-1* promoter [51] and were previously shown to also modulate quinine avoidance [40,43], although the contribution of ASJ appeared to be quite minor [40]. Knocking down *unc-9* in either ASJ using the *trx-1* promoter [61], or ASK using the *srbc-66* promoter [62], resulted in modest hypersensitivity (Figure 3E).

### 3.3. UNC-7 and UNC-9 Locomotor Rescue Facilitates Behavioral Analysis

**Figure 4:**
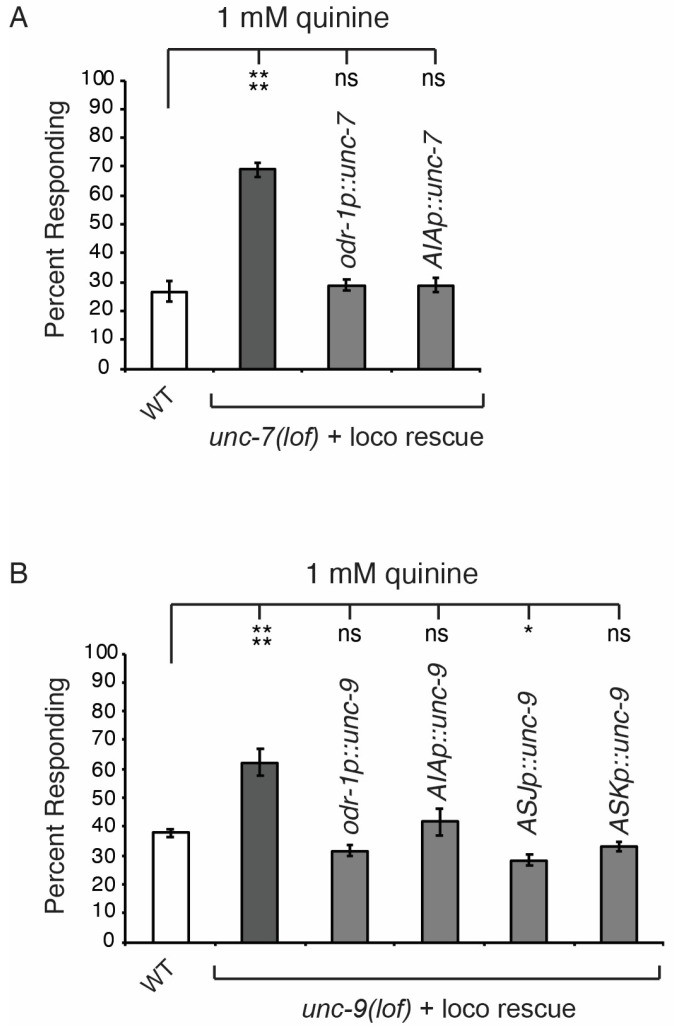
UNC-7 and UNC-9 locomotor rescue facilitates behavioral analysis. (**A**) Expression of *unc-7* using either the *odr-1* promoter (AWB, AWC, ASI, ASJ and ASK) [51] or the *gcy-28d* AIA promoter [53] was sufficient to restore wild-type response to quinine in *unc-7(e5)* loss-of-function animals, when assessed in combination with locomotor rescue using *glr-1p::unc-7* [63,64] (*p* > 0.9 for both). (**B**) Expression of *unc-9* using either the *odr-1* promoter (*p* > 0.3), or cell selective expression using the AIA *gcy-28d* promoter (*p* > 0.8), the ASJ *trx-1* promoter [61] (*p* < 0.04, but the response was slightly less that wild-type animals), or the ASK *srbc-66* promoter [62] (*p* > 0.7) rescued the quinine hypersensitivity of *unc-9(e101)* loss-of-function animals, when assessed in combination with locomotor rescue using *unc-4p::unc-9* [65]. The combined data of ≥3 independent lines and n ≥ 60 transgenic animals are shown. The percentage of animals responding is shown. WT = the N2 wild-type strain. loco = locomotion. lof = loss-of-function. * denotes *p* < 0.05, and **** denotes *p* < 0.0001. ns denotes *p* ≥ 0.05 (not significant).

The RNAi data presented above (Figure 3) strongly suggested a role for UNC-7 and UNC-9 in the regulation of quinine sensitivity. To further confirm a role for these innexins, we sought to also assess cell-specific rescue of each. However, since *unc-7(lof)* and *unc-9(lof)* animals have movement defects [44,59,60], we first needed to rescue their locomotion. Normal movement was restored in *unc-7(lof)* animals via expression in interneurons (*glr-1p::unc-7*)*,* as previously described [63,64], which revealed the quinine hypersensitivity phenotype of *unc-7(lof)* animals (Figure 4A). Consistent with RNAi knockdown (Figure 3C), expression of *unc-7* cDNA using either the *odr-1* promoter or the *gcy-28d* promoter (AIA) in locomotor-rescued animals rescued the quinine hypersensitivity and restored the response to wild-type levels (Figure 4A). Similarly, normal movement was restored in *unc-9(lof)* animals so that we could perform behavioral assays, this time expressing *unc-9* cDNA under the control of the *unc-4* A motor neuron promoter [65], as previously described [63,64]. Locomotor-rescued *unc-9(lof)* animals were also hypersensitive to dilute quinine (Figure 4B). However, *unc-9(lof)* animals with locomotion restored and also expressing *unc-9* cDNA with either the *odr-1* promoter or the *gcy-28d* promoter (AIA) were no longer hypersensitive (Figure 4B). Consistent with the RNAi data (Figure 3E), *unc-9* expression in just ASJ (*trx-1* promoter) or ASK (*srbc-66* promoter) was also sufficient to rescue behavioral hypersensitivity to quinine (Figure 4B).

## 4. Discussion

Behavior is a complex multi-faceted output, resulting from the integration of sensory and modulatory inputs. This integration enables context-appropriate responses to stimuli, reflective of the animal’s current state (e.g., feeding/nutritional status, sleep/wake cycles, emotional input, etc.) [66,67,68,69,70,71]. In *C. elegans*, avoidance of aversive stimuli is modulated by feeding status, with wild-type animals being more sensitive to noxious cues when they are well fed than they are following food deprivation [35,36,37,38,39,40]. While well-fed animals can afford to be wary of environments forewarning potential hazards, if an animal is starving, diminished aversive responses likely allow for entry into more (even potentially dangerous) environments to increase the likelihood of encountering new food sources when an animal’s nutritional needs are high.

The primary nociceptors in *C. elegans* are the ASH sensory neurons. They detect a broad range of noxious stimuli [34,72], including the bitter tastant quinine [23] used throughout the studies presented here. We have previously shown that behavioral sensitivity to ASH-detected noxious stimuli is tuned by several additional head sensory neurons [40] that themselves detect a diverse array of attractive and aversive stimuli [34,72]. These neurons are connected to ASH (indirectly and directly, Supplementary Figure S1) via a gap junction-based neural network that serves to deliver cGMP produced at distant sites to ASH, where it can then dampen sensory signaling and ASH-mediated nociceptive sensitivity [40,41,42,43]. For example, the guanylyl cyclase ODR-1 functions in the AWC sensory neurons to mediate chemotaxis to attractive odorants (indicative of a food source), while it functions in AWB to mediate avoidance of 2-nonanone [51]. AFD is thermosensory [73,74], and the ADL, ASK and ASI neurons are all important for pheromone detection (a proxy for high population density and therefore potentially dwindling food reserves) [34]. Additionally, nutritional status regulates cGMP signaling in ASI [75]. Thus, this gap junction network may allow for the efficient integration of diverse signals to allow for a more sophisticated modulation of behavior than could be achieved by expressing the guanylyl cyclase(s) directly in the ASHs.

**Figure 5:**
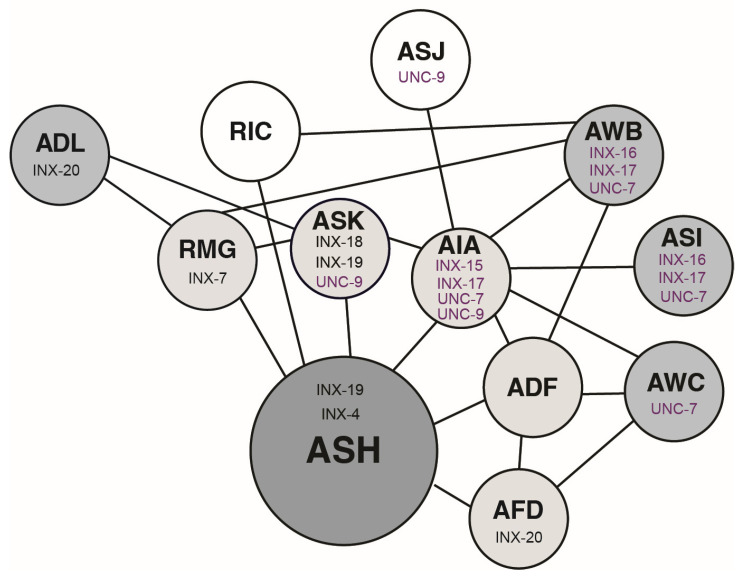
Model of innexin-based gap junction network that modulates ASH behavioral sensitivity. This diagram reflects the re-annotated wiring diagram [77] of the original electron micrograph series [52], as curated at WormWiring.org, accessed on 19 February 2025. Black lines indicate gap junction connections between neurons. Our working model is that cGMP produced by the guanylyl cyclase ODR-1 in AWB, AWC and ASI [40], and a yet unidentified guanylyl cyclase in ADL [43], moves through gap junction connections to the ASH nociceptors. Medium grey indicates neurons that produce cGMP. Light grey indicates neurons through which cGMP may pass. Once in ASH, cGMP activates the cGMP-dependent protein kinase EGL-4, which likely directly phosphorylates the regulator of G protein signaling proteins RGS-2 and RGS-3, stimulating their activity [41] to downregulate Gα proteins. The site of action for each innexin shown to modulate ASH sensitivity is shown: INX-7 [RMG—this study and [43]]; INX-15 (AIA—this study); INX-16 (AWB, ASI—this study); INX-17 (AWB, ASI, AIA—this study); UNC-7 (AWB, AWC, ASI, AIA—this study); UNC-9 (ASJ, ASK, AIA—this study); INX-20 (ADL, AFD—[43]); INX-18 (ASK—[42]); INX-19 (ASK, ASH—[42]); INX-4 (ASH—[40]). Magenta coloration indicates this study’s additions to the modulatory circuit. Figure adapted from Ref. [43].

The *C. elegans* genome encodes 25 innexins, which in unknown combinations can form the 890 gap junctions anatomically defined in the updated wiring diagram of the worm’s nervous system [76]. We previously showed that INX-4 functions in ASH to allow cGMP entry [40], and INX-20 functions in ADL (which is indirectly connected to ASH via RMG and ASK) and AFD (which lies between ASH and additional modulatory neurons) [43]. Additional studies highlighted a role for INX-19 in forming gap junctions between ASK and ASH to pass cGMP to ASH [42]. INX-18 also functions in ASK, but its primary role here may be in stabilizing INX-19-containing electrical synapses [42]. Aside from these few examples, the identity of gap junction components required for passage of cGMP from its sites of production (primarily AWB, AWC, ASI), through intermediary neurons, to the ASHs was unknown. Using a combination of genetic mutants, cell-selective rescue, and cell-selective knockdown experiments, we have identified six additional innexins that regulate ASH-mediated nociceptive sensitivity, as well as their sites of action—INX-7, INX-15, INX-16, INX-17, UNC-7 and UNC-9 (Figure 5). The only neuron shown to function within the modulatory network without a characterized innexin is ADF, although we note that we did not use an ADF-specific promoter in our study.

Although original GFP reporter analysis indicated INX-7 expression in many neurons (as well as cells of the alimentary canal), none were within the ASH modulatory circuit [16]. However, the *C. elegans* Neuronal Gene Expression Map and Network (CeNGEN) single-cell transcriptional profiling project has now revealed *inx-7* expression in each cell of the circuit, with RMG having the third highest expression levels of *inx-7* in the nervous system (https://cengen.shinyapps.io/CengenApp/, accessed on 19 February 2025) [78]. This is consistent with our finding that INX-7 function in RMG is important for regulating ASH-mediated quinine sensitivity (Figure 2) [43]. This is, to our knowledge, the only known in vivo role for INX-7.

Neuronal expression is not currently reported by CeNGEN as detected for *inx-15*, *inx-16*, or *inx-17* (https://cengen.shinyapps.io/CengenApp/, accessed on 19 February 2025) [78]. However, we note that CeNGEN profiled neuronal expression of L4-staged larvae, not adult animals as were used in our behavioral assays. Fosmid reporter expression also did not show neuronal expression for these genes, but it is possible that some innexins might only be expressed at extremely low levels or under certain physiological conditions. However, our cell-specific rescue experiments strongly support a role for INX-15, INX-16 and INX-17 within the modulatory circuit (Figure 2). GFP reporter analysis revealed INX-15 expression exclusively in intestinal cells [16], although its role there is unknown. The genes encoding INX-16 and INX-17 are contained within an operon, separated by just 50 base pairs. Fluorescent reporter analysis showed high levels of INX-16 and weaker levels of INX-17 expressed in intestinal cells [16]. INX-17 was also seen in a few additional cells of the alimentary canal, as well as in HMC (head mesodermal cell) [16]. While this approach did not show INX-16 in the nervous system, INX-17 was detected in the interneuron AIN of adult hermaphrodites [16,79], as well as in the DVA, DVC and PVT interneurons of early larvae [16]. Consistent with its expression in intestinal cells, INX-16 is required for normal calcium wave propagation through the intestine during defecation behavior [80], as well as for propagating the anterior to posterior wave of intestinal cell death (likely by calcium influx) that is associated with the necrotic cell death pathway and organismal death [81]. INX-16 is also important for normal electrical coupling of body-wall muscle cells [82]. However, an in vivo role for INX-17 has not been reported prior to our study.

In addition to reporter expression showing UNC-7 and UNC-9 as being in several non-neuronal cells of the animal [16], among all innexins they (along with INX-7, which frequently co-localized with UNC-9) were the most broadly expressed in the nervous system [16,83]. Supporting the fluorescent reporter analysis, CeNGEN currently shows *unc-7* to be expressed in 86 different neuron types (including ASI), and *unc-9* in 107 (including AIA) (https://cengen.shinyapps.io/CengenApp/, accessed on 19 February 2025) [78]. Fosmid reporter analysis also showed *unc-7* to be expressed in ASI, along with AWB and AWC, and *unc-9* to be expressed in ASJ, ASK and AIA [84]. We do note that *unc-7* expression has not yet been detected in AIA.

*unc-7* and *unc-9* are perhaps best known for their role in locomotion; mutation of either leads to the “uncoordinated” movement phenotype for which the genes are named [44,59,60]. As such, animals harboring genomic loss-of-function mutations in these genes are poor candidates for most behavioral assays, leaving their roles in sensory signaling largely unknown. However, UNC-7 was shown to function, most likely through gap junctions, in the mechanosensory nose touch circuit [85]. Evoked calcium transients are reduced in the FLP mechanosensory neuron, and absent in the RIH interneuron of *unc-7* mutants, and the nose touch escape behavior of these animals could be rescued by *unc-7* expression in the nose touch circuit [85]. In addition, calcium imaging suggested that UNC-7 hemichannels play an important role in gentle touch mechanosensation in the anterior and posterior gentle touch neurons, while both UNC-7 and UNC-9 are required for gap junction communication between anterior gentle touch neurons [86]. UNC-7’s role in regulating thermotaxis behavior was also recently demonstrated in animals lacking *unc-7* in only the AFD thermosensory neurons (generated using the Cre/*loxP* system). Although in this behavior UNC-7 functions as a hemichannel to transmit temperature information from AFD to the AIY interneurons (and possibly the RMD motor neuron) [87].

Our work has revealed new in vivo functions for six innexins. Combined with previous work [40,42,43], an extensive network of possible gap junction connections capable of modulating chemosensory behavior has emerged (Figure 5). For example, within RMG, INX-7 could form gap junctions with INX-20 in ADL, while three innexins function within ASK (INX-18, INX-19, UNC-9) and could interact in either homomeric or heteromeric combinations with RMG-expressed INX-7 (although we note that currently there isn’t functional data to support these various combinations). In the locomotor circuit UNC-7 functions in the AVB forward command interneurons, forming gap junctions with UNC-9 in their downstream partners—B class motor neurons [88]. Thus, it is possible that UNC-7 in AWB/ASI/AWC could interact in this homomeric/heterotypic way with UNC-9 in AIA. Similarly, UNC-7 in AIA could form heterotypic gap junctions with UNC-9 in ASK. UNC-7 could also form homomeric/homotypic channels, as suggested by studies in the male sensory system [89]. As several innexins function in AWB, ASI, ASK and AIA, they could come together in either homomeric or heteromeric combinations—perhaps being recruited and used differently under different physiological conditions, changing the path of cGMP through the circuit as appropriate to integrate diverse sensory signals. However, we cannot rule out the possibility that ectopic gap junctions might have formed in combinations that are not present endogenously, to facilitate rescue in the cell-selective expression experiments.

The non-cell-autonomous regulation of ASH sensitivity by innexins highlights the intricacy with which neural circuits can coordinate to regulate neuronal signaling, allowing informed and appropriate behavioral responses. Given the multiple “paths” through the circuit that cGMP could take to reach ASH, and that several of the neurons use more than one innexin, it is somewhat surprising that loss of a single innexin can result in quinine hypersensitivity. However, this is consistent with our previous finding that ablation of even a single neuron in the circuit can lead to hypersensitivity—although greater hypersensitivity was seen when multiple neurons were ablated simultaneously [40]. Future studies tracking cGMP movement through the circuit and calcium dynamics in ASH, in wild-type and innexin mutant animals, will shed further light on how neurons within the circuit coordinate to modulate ASH activity. More broadly, our work highlights the significance of gap junction components on animal physiology and behavior, beyond their well-defined role in the electrical coupling of cells. Gaining a better understanding of the variety of ways in which gap junctions contribute to and regulate activity within a neural circuit may also be beneficial to characterizing and treating human ailments, as mutations in connexin-encoding genes have been connected to a variety of health concerns, including neurological disorders (e.g., Parkinson’s disease, Alzheimer’s disease and epilepsy), cataracts and cardiopathies [3,90,91,92].

## Data Availability

The raw data supporting the conclusions of this article will be made available by the authors on request.

## Supplementary Materials

The following supporting information can be downloaded at: https://www.mdpi.com/article/10.3390/genes16030260/s1, Figure S1: ASH modulatory network; Table S1: Strains and plasmids.

## References

[1] Simon A.M. (1999). Gap junctions: More roles and new structural data. Trends Cell Biol..

[2] Goldberg G.S., Lampe P.D., Nicholson B.J. (1999). Selective transfer of endogenous metabolites through gap junctions composed of different connexins. Nat. Cell Biol..

[3] Kirchhoff S., Nelles E., Hagendorff A., Kruger O., Traub O., Willecke K. (1998). Reduced cardiac conduction velocity and predisposition to arrhythmias in connexin40-deficient mice. Curr. Biol..

[4] Saez J.C., Connor J.A., Spray D.C., Bennett M.V. (1989). Hepatocyte gap junctions are permeable to the second messenger, inositol 1,4,5-trisphosphate, and to calcium ions. Proc. Natl. Acad. Sci. USA.

[5] Anderson E., Albertini D.F. (1976). Gap junctions between the oocyte and companion follicle cells in the mammalian ovary. J. Cell Biol..

[6] Mao G.K., Li J.X., Bian F.H., Han Y.Y., Guo M., Xu B.S., Zhang M.J., Xia G.L. (2013). Gap junction-mediated cAMP movement between oocytes and somatic cells. Front. Biosci. (Elite Ed.).

[7] Norris R.P., Ratzan W.J., Freudzon M., Mehlmann L.M., Krall J., Movsesian M.A., Wang H., Ke H., Nikolaev V.O., Jaffe L.A. (2009). Cyclic GMP from the surrounding somatic cells regulates cyclic AMP and meiosis in the mouse oocyte. Development.

[8] Shuhaibar L.C., Egbert J.R., Norris R.P., Lampe P.D., Nikolaev V.O., Thunemann M., Wen L., Feil R., Jaffe L.A. (2015). Intercellular signaling via cyclic GMP diffusion through gap junctions restarts meiosis in mouse ovarian follicles. Proc. Natl. Acad. Sci. USA.

[9] Vaccari S., Weeks J.L., Hsieh M., Menniti F.S., Conti M. (2009). Cyclic GMP signaling is involved in the luteinizing hormone-dependent meiotic maturation of mouse oocytes. Biol. Reprod..

[10] Beyer E.C., Berthoud V.M. (2018). Gap junction gene and protein families: Connexins, innexins, and pannexins. Biochim. Biophys. Acta Biomembr..

[11] Skerrett I.M., Williams J.B. (2017). A structural and functional comparison of gap junction channels composed of connexins and innexins. Dev. Neurobiol..

[12] Phelan P., Starich T.A. (2001). Innexins get into the gap. Bioessays.

[13] Sohl G., Maxeiner S., Willecke K. (2005). Expression and functions of neuronal gap junctions. Nat. Rev. Neurosci..

[14] Koval M., Molina S.A., Burt J.M. (2014). Mix and match: Investigating heteromeric and heterotypic gap junction channels in model systems and native tissues. FEBS Lett..

[15] Sosinsky G.E., Nicholson B.J. (2005). Structural organization of gap junction channels. Biochim. Biophys. Acta.

[16] Altun Z.F., Chen B., Wang Z.W., Hall D.H. (2009). High resolution map of *Caenorhabditis elegans* gap junction proteins. Dev Dyn.

[17] Jin E.J., Park S., Lyu X., Jin Y. (2020). Gap junctions: Historical discoveries and new findings in the *Caenorhabditis elegans* nervous system. Biol. Open.

[18] Bargmann C.I., Thomas J.H., Horvitz H.R. (1990). Chemosensory cell function in the behavior and development of *Caenorhabditis elegans*. Cold Spring Harb. Symp. Quant. Biol..

[19] Chatzigeorgiou M., Bang S., Hwang S.W., Schafer W.R. (2013). *tmc-1* encodes a sodium-sensitive channel required for salt chemosensation in *C. elegans*. Nature.

[20] Hart A.C., Kass J., Shapiro J.E., Kaplan J.M. (1999). Distinct signaling pathways mediate touch and osmosensory responses in a polymodal sensory neuron. J. Neurosci..

[21] Hilliard M.A., Apicella A.J., Kerr R., Suzuki H., Bazzicalupo P., Schafer W.R. (2005). In vivo imaging of *C. elegans* ASH neurons: Cellular response and adaptation to chemical repellents. EMBO J..

[22] Hilliard M.A., Bargmann C.I., Bazzicalupo P.C. (2002). *elegans* responds to chemical repellents by integrating sensory inputs from the head and the tail. Curr. Biol..

[23] Hilliard M.A., Bergamasco C., Arbucci S., Plasterk R.H., Bazzicalupo P. (2004). Worms taste bitter: ASH neurons, QUI-1, GPA-3 and ODR-3 mediate quinine avoidance in *Caenorhabditis elegans*. EMBO J..

[24] Kaplan J.M., Horvitz H.R. (1993). A dual mechanosensory and chemosensory neuron in *Caenorhabditis elegans*. Proc. Natl. Acad. Sci. USA.

[25] Liu Z., Kariya M.J., Chute C.D., Pribadi A.K., Leinwand S.G., Tong A., Curran K.P., Bose N., Schroeder F.C., Srinivasan J. (2018). Predator-secreted sulfolipids induce defensive responses in *C. elegans*. Nat. Commun..

[26] Sambongi Y., Nagae T., Liu Y., Yoshimizu T., Takeda K., Wada Y., Futai M. (1999). Sensing of cadmium and copper ions by externally exposed ADL, ASE, and ASH neurons elicits avoidance response in *Caenorhabditis elegans*. Neuroreport.

[27] Troemel E.R. (1999). Chemosensory signaling in *C. elegans*. Bioessays.

[28] Sambongi Y., Takeda K., Wakabayashi T., Ueda I., Wada Y., Futai M. (2000). *Caenorhabditis elegans* senses protons through amphid chemosensory neurons: Proton signals elicit avoidance behavior. Neuroreport.

[29] Sassa T., Maruyama I.N. (2013). A G-protein α subunit, GOA-1, plays a role in *C. elegans* avoidance behavior of strongly alkaline pH. Commun. Integr. Biol..

[30] Taniguchi G., Uozumi T., Kiriyama K., Kamizaki T., Hirotsu T. (2014). Screening of odor-receptor pairs in *Caenorhabditis elegans* reveals different receptors for high and low odor concentrations. Sci. Signal..

[31] Tran A., Tang A., O’Loughlin C.T., Balistreri A., Chang E., Coto Villa D., Li J., Varshney A., Jimenez V., Pyle J. (2017). *C. elegans* avoids toxin-producing *Streptomyces* using a seven transmembrane domain chemosensory receptor. eLife.

[32] Troemel E.R., Chou J.H., Dwyer N.D., Colbert H.A., Bargmann C.I. (1995). Divergent seven transmembrane receptors are candidate chemosensory receptors in *C. elegans*. Cell.

[33] Yoshida K., Hirotsu T., Tagawa T., Oda S., Wakabayashi T., Iino Y., Ishihara T. (2012). Odour concentration-dependent olfactory preference change in *C. elegans*. Nat. Commun..

[34] Ferkey D.M., Sengupta P., L’Etoile N.D. (2021). Chemosensory signal transduction in *Caenorhabditis elegans*. Genetics.

[35] Chao M.Y., Komatsu H., Fukuto H.S., Dionne H.M., Hart A.C. (2004). Feeding status and serotonin rapidly and reversibly modulate a *Caenorhabditis elegans* chemosensory circuit. Proc. Natl. Acad. Sci. USA.

[36] Ferkey D.M., Hyde R., Haspel G., Dionne H.M., Hess H.A., Suzuki H., Schafer W.R., Koelle M.R., Hart A.C. (2007). *C. elegans* G protein regulator RGS-3 controls sensitivity to sensory stimuli. Neuron.

[37] Wragg R.T., Hapiak V., Miller S.B., Harris G.P., Gray J., Komuniecki P.R., Komuniecki R.W. (2007). Tyramine and octopamine independently inhibit serotonin-stimulated aversive behaviors in *Caenorhabditis elegans* through two novel amine receptors. J. Neurosci..

[38] Harris G.P., Hapiak V.M., Wragg R.T., Miller S.B., Hughes L.J., Hobson R.J., Steven R., Bamber B., Komuniecki R.W. (2009). Three distinct amine receptors operating at different levels within the locomotory circuit are each essential for the serotonergic modulation of chemosensation in *Caenorhabditis elegans*. J. Neurosci..

[39] Ezcurra M., Tanizawa Y., Swoboda P., Schafer W.R. (2011). Food sensitizes *C. elegans* avoidance behaviours through acute dopamine signalling. EMBO J..

[40] Krzyzanowski M.C., Woldemariam S., Wood J.F., Chaubey A.H., Brueggemann C., Bowitch A., Bethke M., L’Etoile N.D., Ferkey D.M. (2016). Aversive Behavior in the Nematode *C. elegans* is Modulated by cGMP and a Neuronal Gap Junction Network. PLoS Genet..

[41] Krzyzanowski M.C., Brueggemann C., Ezak M.J., Wood J.F., Michaels K.L., Jackson C.A., Juang B.T., Collins K.D., Yu M.C., L’Etoile N.D. (2013). The *C. elegans* cGMP-dependent protein kinase EGL-4 regulates nociceptive behavioral sensitivity. PLoS Genet..

[42] Voelker L., Upadhyaya B., Ferkey D.M., Woldemariam S., L’Etoile N.D., Rabinowitch I., Bai J. (2019). INX-18 and INX-19 play distinct roles in electrical synapses that modulate aversive behavior in *Caenorhabditis elegans*. PLoS Genet..

[43] Chaubey A.H., Sojka S.E., Onukwufor J.O., Ezak M.J., Vandermeulen M.D., Bowitch A., Vodickova A., Wojtovich A.P., Ferkey D.M. (2023). The *Caenorhabditis elegans* innexin INX-20 regulates nociceptive behavioral sensitivity. Genetics.

[44] Brenner S. (1974). The genetics of *Caenorhabditis elegans*. Genetics.

[45] Fukuto H.S., Ferkey D.M., Apicella A.J., Lans H., Sharmeen T., Chen W., Lefkowitz R.J., Jansen G., Schafer W.R., Hart A.C. (2004). G protein-coupled receptor kinase function is essential for chemosensation in *C. elegans*. Neuron.

[46] Ezak M.J., Hong E., Chaparro-Garcia A., Ferkey D.M. (2010). *Caenorhabditis elegans* TRPV channels function in a modality-specific pathway to regulate response to aberrant sensory signaling. Genetics.

[47] Wood W.B. (1988). The Nematode Caenorhabditis elegans.

[48] Esposito G., Di Schiavi E., Bergamasco C., Bazzicalupo P. (2007). Efficient and cell specific knock-down of gene function in targeted *C. elegans* neurons. Gene.

[49] Fukushige T., Hawkins M.G., McGhee J.D. (1998). The GATA-factor *elt-2* is essential for formation of the *Caenorhabditis elegans* intestine. Dev. Biol..

[50] Mello C.C., Kramer J.M., Stinchcomb D., Ambros V. (1991). Efficient gene transfer in *C.elegans*: Extrachromosomal maintenance and integration of transforming sequences. EMBO J..

[51] L’Etoile N.D., Bargmann C.I. (2000). Olfaction and odor discrimination are mediated by the *C. elegans* guanylyl cyclase ODR-1. Neuron.

[52] White J.G., Southgate E., Thomson J.N., Brenner S. (1986). The structure of the nervous system of the nematode *Caenorhabditis elegans*. Philos. Trans. R. Soc. Lond. B Biol. Sci..

[53] Shinkai Y., Yamamoto Y., Fujiwara M., Tabata T., Murayama T., Hirotsu T., Ikeda D.D., Tsunozaki M., Iino Y., Bargmann C.I. (2011). Behavioral choice between conflicting alternatives is regulated by a receptor guanylyl cyclase, GCY-28, and a receptor tyrosine kinase, SCD-2, in AIA interneurons of *Caenorhabditis elegans*. J. Neurosci..

[54] Taylor S.R., Santpere G., Reilly M., Glenwinkel L., Poff A., McWhirter R., Xu C., Weinreb A., Basavaraju M., Cook S.J. (2019). Expression profiling of the mature *C. elegans* nervous system by single-cell RNA-Sequencing. bioRxiv.

[55] Lorenzo R., Onizuka M., Defrance M., Laurent P. (2020). Combining single-cell RNA-sequencing with a molecular atlas unveils new markers for *Caenorhabditis elegans* neuron classes. Nucleic Acids Res..

[56] Troemel E.R., Kimmel B.E., Bargmann C.I. (1997). Reprogramming chemotaxis responses: Sensory neurons define olfactory preferences in *C. elegans*. Cell.

[57] Etchberger J.F., Lorch A., Sleumer M.C., Zapf R., Jones S.J., Marra M.A., Holt R.A., Moerman D.G., Hobert O. (2007). The molecular signature and cis-regulatory architecture of a *C. elegans* gustatory neuron. Genes Dev..

[58] Jansen G., Thijssen K.L., Werner P., van der Horst M., Hazendonk E., Plasterk R.H. (1999). The complete family of genes encoding G proteins of *Caenorhabditis elegans*. Nat. Genet..

[59] Starich T.A., Herman R.K., Shaw J.E. (1993). Molecular and genetic analysis of *unc-7*, a *Caenorhabditis elegans* gene required for coordinated locomotion. Genetics.

[60] Barnes T.M., Hekimi S. (1997). The *Caenorhabditis elegans* avermectin resistance and anesthetic response gene *unc-9* encodes a member of a protein family implicated in electrical coupling of excitable cells. J. Neurochem..

[61] Miranda-Vizuete A., Fierro Gonzalez J.C., Gahmon G., Burghoorn J., Navas P., Swoboda P. (2006). Lifespan decrease in a *Caenorhabditis elegans* mutant lacking TRX-1, a thioredoxin expressed in ASJ sensory neurons. FEBS Lett..

[62] Kim K., Sato K., Shibuya M., Zeiger D.M., Butcher R.A., Ragains J.R., Clardy J., Touhara K., Sengupta P. (2009). Two chemoreceptors mediate developmental effects of dauer pheromone in *C. elegans*. Science.

[63] Huang H., Hayden D.J., Zhu C.T., Bennett H.L., Venkatachalam V., Skuja L.L., Hart A.C. (2018). Gap Junctions and NCA Cation Channels Are Critical for Developmentally Timed Sleep and Arousal in *Caenorhabditis elegans*. Genetics.

[64] Kawano T., Po M.D., Gao S., Leung G., Ryu W.S., Zhen M. (2011). An imbalancing act: Gap junctions reduce the backward motor circuit activity to bias *C. elegans* for forward locomotion. Neuron.

[65] Miller D.M., Shen M.M., Shamu C.E., Burglin T.R., Ruvkun G., Dubois M.L., Ghee M., Wilson L. (1992). *C. elegans unc-4* gene encodes a homeodomain protein that determines the pattern of synaptic input to specific motor neurons. Nature.

[66] Shanahan L.K., Kahnt T. (2022). On the state-dependent nature of odor perception. Front. Neurosci..

[67] Sengupta P. (2013). The belly rules the nose: Feeding state-dependent modulation of peripheral chemosensory responses. Curr. Opin. Neurobiol..

[68] Gaeta G., Wilson D.A. (2022). Reciprocal relationships between sleep and smell. Front. Neural Circuits.

[69] Yamaguchi M. (2017). The role of sleep in the plasticity of the olfactory system. Neurosci. Res..

[70] Kontaris I., East B.S., Wilson D.A. (2020). Behavioral and Neurobiological Convergence of Odor, Mood and Emotion: A Review. Front. Behav. Neurosci..

[71] Baumeister R.F., Vohs K.D., DeWall C.N., Zhang L. (2007). How emotion shapes behavior: Feedback, anticipation, and reflection, rather than direct causation. Pers. Soc. Psychol. Rev..

[72] Bargmann C.I. Chemosensation in *C. elegans*. http://www.wormbook.org/chapters/www_chemosensation/chemosensation.html.

[73] Goodman M.B., Sengupta P. (2019). How *Caenorhabditis elegans* Senses Mechanical Stress, Temperature, and Other Physical Stimuli. Genetics.

[74] Goodman M.B., Sengupta P. (2018). The extraordinary AFD thermosensor of *C. elegans*. Pflug. Arch..

[75] Gallagher T., Kim J., Oldenbroek M., Kerr R., You Y.J. (2013). ASI regulates satiety quiescence in *C. elegans*. J. Neurosci..

[76] Varshney L.R., Chen B.L., Paniagua E., Hall D.H., Chklovskii D.B. (2011). Structural properties of the *Caenorhabditis elegans* neuronal network. PLoS Comput. Biol..

[77] Cook S.J., Jarrell T.A., Brittin C.A., Wang Y., Bloniarz A.E., Yakovlev M.A., Nguyen K.C.Q., Tang L.T., Bayer E.A., Duerr J.S. (2019). Whole-animal connectomes of both *Caenorhabditis elegans* sexes. Nature.

[78] Hammarlund M., Hobert O., Miller D.M., Sestan N. (2018). The CeNGEN Project: The Complete Gene Expression Map of an Entire Nervous System. Neuron.

[79] Takagaki N., Ohta A., Ohnishi K., Kawanabe A., Minakuchi Y., Toyoda A., Fujiwara Y., Kuhara A. (2020). The mechanoreceptor DEG-1 regulates cold tolerance in *Caenorhabditis elegans*. EMBO Rep..

[80] Peters M.A., Teramoto T., White J.Q., Iwasaki K., Jorgensen E.M. (2007). A calcium wave mediated by gap junctions coordinates a rhythmic behavior in *C. elegans*. Curr. Biol..

[81] Coburn C., Allman E., Mahanti P., Benedetto A., Cabreiro F., Pincus Z., Matthijssens F., Araiz C., Mandel A., Vlachos M. (2013). Anthranilate fluorescence marks a calcium-propagated necrotic wave that promotes organismal death in *C. elegans*. PLoS Biol..

[82] Liu P., Chen B., Altun Z.F., Gross M.J., Shan A., Schuman B., Hall D.H., Wang Z.W. (2013). Six innexins contribute to electrical coupling of *C. elegans* body-wall muscle. PLoS ONE.

[83] Starich T., Sheehan M., Jadrich J., Shaw J. (2001). Innexins in *C. elegans*. Cell Commun. Adhes..

[84] Bhattacharya A., Aghayeva U., Berghoff E.G., Hobert O. (2019). Plasticity of the Electrical Connectome of *C. elegans*. Cell.

[85] Chatzigeorgiou M., Schafer W.R. (2011). Lateral facilitation between primary mechanosensory neurons controls nose touch perception in *C. elegans*. Neuron.

[86] Walker D.S., Schafer W.R. (2020). Distinct roles for innexin gap junctions and hemichannels in mechanosensation. eLife.

[87] Nakayama A., Watanabe M., Yamashiro R., Kuroyanagi H., Matsuyama H.J., Oshima A., Mori I., Nakano S. (2024). A hyperpolarizing neuron recruits undocked innexin hemichannels to transmit neural information in *Caenorhabditis elegans*. Proc. Natl. Acad. Sci. USA.

[88] Starich T.A., Xu J., Skerrett I.M., Nicholson B.J., Shaw J.E. (2009). Interactions between innexins UNC-7 and UNC-9 mediate electrical synapse specificity in the *Caenorhabditis elegans* locomotory nervous system. Neural Dev..

[89] Correa P.A., Gruninger T., Garcia L.R. (2015). DOP-2 D2-Like Receptor Regulates UNC-7 Innexins to Attenuate Recurrent Sensory Motor Neurons during *C. elegans* Copulation. J. Neurosci..

[90] Beyer E.C., Ebihara L., Berthoud V.M. (2013). Connexin mutants and cataracts. Front. Pharmacol..

[91] Choudhury S.P., Bano S., Sen S., Suchal K., Kumar S., Nikolajeff F., Dey S.K., Sharma V. (2022). Altered neural cell junctions and ion-channels leading to disrupted neuron communication in Parkinson’s disease. npj Parkinsons Dis..

[92] Nakase T., Naus C.C. (2004). Gap junctions and neurological disorders of the central nervous system. Biochim. Biophys. Acta.

