# Supplementary material for: An Extensive Gap Junction Neural Network Modulates Caenorhabditis elegans Aversive Behavior
Source: Genes (Basel). 2025 Feb 23;16(3):260. doi: 10.3390/genes16030260 (PMC11941935; doi:10.3390/genes16030260)
Supplement: Supplementary file 1 [file genes-16-00260-s001.zip › genes-3488669-supplementary.pdf]

Supplemental Information:

Table S1: Strains and plasmids

CGC = *Caenorhabditis* Genetics Center
NRBP = National BioResource Project

Name	Genotype	Source
N2	Bristol wild-type	CGC
FG613	*inx-20(ok426)* 5X outcross	[1]
**Loss-of-function innexin alleles**		
FX18538	*inx-1(tm3524)*	NRBP
FG928	*inx-1(tm3524)* 5X outcross	This study
FX18537	*inx-1(tm6662)*	NRBP
FG927	*inx-1(tm6662)* 5X outcross	This study
RB1792	*inx-7(ok2319)*	CGC
FG1120	*inx-7(ok2319)* 5X outcross	This study
FX03394	*inx-15(tm3394)*	NRBP
FG1084	*inx-15(tm3394)* 5X outcross	This study
EG144	*inx-16(ox144)* 3X outcross	CGC
FX03839	*inx-17(tm3839)*	NRBP
FG982	*inx-17(tm3839)* 5X outcross	This study
FX03292	*inx-17(tm3292)*	NRBP
FG929	*inx-17(tm3292)* 5X outcross	This study
CB5	*unc-7(e5)*	CGC
FG981	*unc-7(e5)* 5X outcross	This study
CB101	*unc-9(e101)*	CGC

***inx-7* Rescue Experiments**		
FG1124	*inx-7(ok2319); udEx696[odr-1p::inx-7* (cDNA) + elt-2::gfp]	This study
FG1125	*inx-7(ok2319); udEx697[odr-1p::inx-7* (cDNA) + elt-2::gfp]	This study
FG1126	*inx-7(ok2319); udEx698[odr-1p::inx-7* (cDNA) + elt-2::gfp]	This study
FG1136	*inx-7(ok2319); udE706[gcy-28dp::inx-7* (cDNA) + elt-2::gfp]	This study
FG1137	*inx-7(ok2319); udE707[gcy-28dp::inx-7* (cDNA) + elt-2::gfp]	This study
FG1138	*inx-7(ok2319); udE708[gcy-28dp::inx-7* (cDNA) + elt-2::gfp]	This study
FG1151	*inx-7(ok2319); udE720[nlp-56p::inx-7* (cDNA) + elt-2::gfp]	This study
FG1152	*inx-7(ok2319); udE721[nlp-56p::inx-7* (cDNA) + elt-2::gfp]	This study
FG1153	*inx-7(ok2319); udE722[nlp-56p::inx-7* (cDNA) + elt-2::gfp]	This study
***inx-15* Rescue Experiments**		
FG1127	*inx-15(tm3394); udEx699[odr-1p::inx-15 (cDNA) + elt-2::gfp]*	This study
FG1128	*inx-15(tm3394); udEx700[odr-1p::inx-15 (cDNA) + elt-2::gfp]*	This study
FG1129	*inx-15(tm3394); udEx701[odr-1p::inx-15 (cDNA) + elt-2::gfp]*	This study
FG1130	*inx-15(tm3394); udEx702[gcy-28dp::inx-15 (cDNA) + elt-2::gfp]*	This study
FG1131	*inx-15(tm3394); udEx703[gcy-28dp::inx-15 (cDNA) + elt-2::gfp]*	This study
FG1132	*inx-15(tm3394); udEx704[gcy-28dp::inx-15 (cDNA) + elt-2::gfp]*	This study
FG1133	*inx-15(tm3394); udEx705[gcy-28dp::inx-15 (cDNA) + elt-2::gfp]*	This study
***inx-16* Rescue Experiments**		
FG978	*inx-16(ox144); udEx584[odr-1p::inx-16 (cDNA) + elt-2::gfp]*	This study
FG979	*inx-16(ox144); udEx585[odr-1p::inx-16 (cDNA) + elt-2::gfp]*	This study
FG980	*inx-16(ox144); udEx586[odr-1p::inx-16 (cDNA) + elt-2::gfp]*	This study
FG1378	*inx-16(ox144); udEx797[gcy-28dp::inx-16 (cDNA) + elt-2::gfp]*	This study
FG1379	*inx-16(ox144); udEx798[gcy-28dp::inx-16 (cDNA) + elt-2::gfp]*	This study
FG1380	*inx-16(ox144); udEx799[gcy-28dp::inx-16 (cDNA) + elt-2::gfp]*	This study

FG1108	*inx-16(ox144); udEx684[str-1p::inx-16 (cDNA) + elt-2::gfp]*	This study
FG1109	*inx-16(ox144); udEx685[str-1p::inx-16 (cDNA) + elt-2::gfp]*	This study
FG1110	*inx-16(ox144); udEx686[str-1p::inx-16 (cDNA) + elt-2::gfp]*	This study
FG1094	*inx-16(ox144); udEx674[ceh-36p3::inx-16 (cDNA) + elt-2::gfp]*	This study
FG1095	*inx-16(ox144); udEx675[ceh-36p3::inx-16 (cDNA) + elt-2::gfp]*	This study
FG1096	*inx-16(ox144); udEx676[ceh-36p3::inx-16 (cDNA) + elt-2::gfp]*	This study
FG1097	*inx-16(ox144); udEx677[ceh-36p3::inx-16 (cDNA) + elt-2::gfp]*	This study
FG1111	*inx-16(ox144); udEx687[gpa-4p::inx-16 (cDNA) + elt-2::gfp]*	This study
FG1112	*inx-16(ox144); udEx688[gpa-4p::inx-16 (cDNA) + elt-2::gfp]*	This study
FG1113	*inx-16(ox144); udEx689[gpa-4p::inx-16 (cDNA) + elt-2::gfp]*	This study
***inx-17* Rescue Experiments**		
FG989	*inx-17(tm3292); udEx609[odr-1p::inx-17a (cDNA) + elt-2::gfp]*	This study
FG990	*inx-17(tm3292); udEx610[odr-1p::inx-17a (cDNA) + elt-2::gfp]*	This study
FG991	*inx-17(tm3292); udEx611[odr-1p::inx-17a (cDNA) + elt-2::gfp]*	This study
FG992	*inx-17(tm3292); udEx612[odr-1p::inx-17a (cDNA) + elt-2::gfp]*	This study
FG993	*inx-17(tm3292); udEx613[odr-1p::inx-17a (cDNA) + elt-2::gfp]*	This study
FG994	*inx-17(tm3292); udEx614[odr-1p::inx-17b (cDNA) + elt-2::gfp]*	This study
FG995	*inx-17(tm3292); udEx615[odr-1p::inx-17b (cDNA) + elt-2::gfp]*	This study
FG996	*inx-17(tm3292); udEx616[odr-1p::inx-17b (cDNA) + elt-2::gfp]*	This study
FG997	*inx-17(tm3292); udEx617[gcy-28dp::inx-17a (cDNA) + elt-2::gfp]*	This study
FG998	*inx-17(tm3292); udEx618[gcy-28dp::inx-17a (cDNA) + elt-2::gfp]*	This study
FG999	*inx-17(tm3292); udEx619[gcy-28dp::inx-17a (cDNA) + elt-2::gfp]*	This study
FG1000	*inx-17(tm3292); udEx620[gcy-28dp::inx-17b (cDNA) + elt-2::gfp]*	This study
FG1001	*inx-17(tm3292); udEx621[gcy-28dp::inx-17b (cDNA) + elt-2::gfp]*	This study
FG1002	*inx-17(tm3292); udEx622[gcy-28dp::inx-17b (cDNA) + elt-2::gfp]*	This study

FG1098	*inx-17(tm3292); udEx678[str-1p::inx-17a (cDNA) + elt-2::gfp]*	This study
FG1099	*inx-17(tm3292); udEx679[str-1p::inx-17a (cDNA) + elt-2::gfp]*	This study
FG1100	*inx-17(tm3292); udEx680[str-1p::inx-17a (cDNA) + elt-2::gfp]*	This study
FG1101	*inx-17(tm3292); udEx681[str-1p::inx-17b (cDNA) + elt-2::gfp]*	This study
FG1102	*inx-17(tm3292); udEx682[str-1p::inx-17b (cDNA) + elt-2::gfp]*	This study
FG1103	*inx-17(tm3292); udEx683[str-1p::inx-17b (cDNA) + elt-2::gfp]*	This study
FG1018	*inx-17(tm3292); udEx638[ceh-36p3::inx-17a (cDNA) + elt-2::gfp]*	This study
FG1019	*inx-17(tm3292); udEx639[ceh-36p3::inx-17a (cDNA) + elt-2::gfp]*	This study
FG1020	*inx-17(tm3292); udEx640[ceh-36p3::inx-17a (cDNA) + elt-2::gfp]*	This study
FG1021	*inx-17(tm3292); udEx641[ceh-36p3::inx-17b (cDNA) + elt-2::gfp]*	This study
FG1022	*inx-17(tm3292); udEx642[ceh-36p3::inx-17b (cDNA) + elt-2::gfp]*	This study
FG1023	*inx-17(tm3292); udEx643[ceh-36p3::inx-17b (cDNA) + elt-2::gfp]*	This study
FG1003	*inx-17(tm3292); udEx623[gpa-4p::inx-17a (cDNA) + elt-2::gfp]*	This study
FG1004	*inx-17(tm3292); udEx624[gpa-4p::inx-17a (cDNA) + elt-2::gfp]*	This study
FG1005	*inx-17(tm3292); udEx625[gpa-4p::inx-17a (cDNA) + elt-2::gfp]*	This study
FG1006	*inx-17(tm3292); udEx626[gpa-4p::inx-17b (cDNA) + elt-2::gfp]*	This study
FG1007	*inx-17(tm3292); udEx627[gpa-4p::inx-17b (cDNA) + elt-2::gfp]*	This study
FG1008	*inx-17(tm3292); udEx628[gpa-4p::inx-17b (cDNA) + elt-2::gfp]*	This study
**Innexin RNAi**		
FG881	N2*; udEx509[odr-1p::inx-3 (RNAi) + elt-2::gfp]*	This study
FG882	N2*; udEx510[odr-1p::inx-3 (RNAi) + elt-2::gfp]*	This study
FG883	N2*; udEx511[odr-1p::inx-3 (RNAi) + elt-2::gfp]*	This study
FG884	N2*; udEx512[odr-1p::inx-3 (RNAi) + elt-2::gfp]*	This study
FG885	N2*; udEx513 [odr-1p::inx-12 (RNAi) + elt-2::gfp]*	This study
FG886	N2*; udEx514 [odr-1p::inx-12 (RNAi) + elt-2::gfp]*	This study

FG887	N2*; udEx515 [odr-1p::inx-12 (RNAi) + elt-2::gfp]*	This study
FG888	N2*; udEx516 [odr-1p::inx-13 (RNAi) + elt-2::gfp]*	This study
FG889	N2*; udEx517 [odr-1p::inx-13 (RNAi) + elt-2::gfp]*	This study
FG890	N2*; udEx518 [odr-1p::inx-13 (RNAi) + elt-2::gfp]*	This study
FG1367	N2*; udEx786 [gcy-28dp::inx-3 (RNAi) + elt-2::gfp]*	This study
FG1368	N2*; udEx787 [gcy-28dp::inx-3 (RNAi) + elt-2::gfp]*	This study
FG1369	N2*; udEx788 [gcy-28dp::inx-3 (RNAi) + elt-2::gfp]*	This study
FG1370	N2*; udEx789 [gcy-28dp::inx-12 (RNAi) + elt-2::gfp]*	This study
FG1371	N2*; udEx790 [gcy-28dp::inx-12 (RNAi) + elt-2::gfp]*	This study
FG1372	N2*; udEx791 [gcy-28dp::inx-12 (RNAi) + elt-2::gfp]*	This study
FG1373	N2*; udEx792 [gcy-28dp::inx-13 (RNAi) + elt-2::gfp]*	This study
FG1374	N2*; udEx793 [gcy-28dp::inx-13 (RNAi) + elt-2::gfp]*	This study
FG1375	N2*; udEx794[gcy-28dp::inx-13 (RNAi) + elt-2::gfp]*	This study
FG901	N2; *udEx529[odr-1p::unc-9 (RNAi) + elt-2::gfp]*	This study
FG902	N2; *udEx530[odr-1p::unc-9 (RNAi) + elt-2::gfp]*	This study
FG903	N2; *udEx531[odr-1p::unc-9 (RNAi) + elt-2::gfp]*	This study
FG904	N2; *udEx532[ceh-36p3::unc-9 (RNAi) + elt-2::gfp]*	This study
FG905	N2; *udEx533[ceh-36p3::unc-9 (RNAi) + elt-2::gfp]*	This study
FG906	N2; *udEx534[ceh-36p3::unc-9 (RNAi) + elt-2::gfp]*	This study
FG907	N2; *udEx535[gpa-4p::unc-9 (RNAi) + elt-2::gfp]*	This study
FG908	N2; *udEx536[gpa-4p::unc-9 (RNAi) + elt-2::gfp]*	This study
FG909	N2; *udEx537[gpa-4p::unc-9 (RNAi) + elt-2::gfp]*	This study
FG910	N2; *udEx538[str-1p::unc-9 (RNAi) + elt-2::gfp]*	This study
FG911	N2; *udEx539[str-1p::unc-9 (RNAi) + elt-2::gfp]*	This study
FG912	N2; *udEx540[str-1p::unc-9 (RNAi) + elt-2::gfp]*	This study

FG934	N2; *udEx558[srbc-66p::unc-9 (RNAi) + elt-2::gfp]*	This study
FG935	N2; *udEx559[srbc-66p::unc-9 (RNAi) + elt-2::gfp]*	This study
FG936	N2; *udEx560[srbc-66p::unc-9 (RNAi) + elt-2::gfp]*	This study
FG931	N2; *udEx555[trx-1p::unc-9 (RNAi) + elt-2::gfp]*	This study
FG932	N2; *udEx556[trx-1p::unc-9 (RNAi) + elt-2::gfp]*	This study
FG933	N2; *udEx557[trx-1p::unc-9 (RNAi) + elt-2::gfp]*	This study
FG860	N2; *udEx504[odr-1p::unc-7 (RNAi) + elt-2::gfp]*	This study
FG861	N2; *udEx505[odr-1p::unc-7 (RNAi) + elt-2::gfp]*	This study
FG862	N2; *udEx506[odr-1p::unc-7 (RNAi) + elt-2::gfp]*	This study
FG856	N2; *udEx500[ceh-36p3::unc-7 (RNAi) + elt-2::gfp]*	This study
FG857	N2; *udEx501[ceh-36p3::unc-7 (RNAi) + elt-2::gfp]*	This study
FG858	N2; *udEx502[ceh-36p3::unc-7 (RNAi) + elt-2::gfp]*	This study
FG859	N2; *udEx503[ceh-36p3::unc-7 (RNAi) + elt-2::gfp]*	This study
FG919	N2; *udEx547[gpa-4p::unc-7 (RNAi) + elt-2::gfp]*	This study
FG1376	N2; *udEx795[gpa-4p::unc-7 (RNAi) + elt-2::gfp]*	This study
FG1377	N2; *udEx796[gpa-4p::unc-7 (RNAi) + elt-2::gfp]*	This study
FG913	N2; *udEx541[str-1p::unc-7 (RNAi) + elt-2::gfp]*	This study
FG914	N2; *udEx542[str-1p::unc-7 (RNAi) + elt-2::gfp]*	This study
FG915	N2; *udEx543[str-1p::unc-7 (RNAi) + elt-2::gfp]*	This study
FG916	N2; *udEx544[str-1p::unc-7 (RNAi) + elt-2::gfp]*	This study
FG917	N2; *udEx545[str-1p::unc-7 (RNAi) + elt-2::gfp]*	This study
FG918	N2; *udEx546[str-1p::unc-7 (RNAi) + elt-2::gfp]*	This study
***unc-7* Locomotor and Behavioral Rescues**		
FG1147	*unc-7(e5); udEx717[glr-1p::unc-7 + elt-2::gfp]*	This study
FG1148	*unc-7(e5); udEx718[glr-1p::unc-7 + elt-2::gfp]*	This study

FG1149	*unc-7(e5); udEx719[glr-1p::unc-7 + elt-2::gfp]*	This study
FG1154	*unc-7(e5); udEx723[glr-1p::unc-7 + odr-1p::unc-7 + elt-2::gfp]*	This study
FG1155	*unc-7(e5); udEx724[glr-1p::unc-7 + odr-1p::unc-7 + elt-2::gfp]*	This study
FG1156	*unc-7(e5); udEx725[glr-1p::unc-7 + odr-1p::unc-7 + elt-2::gfp]*	This study
FG1170	*unc-7(e5); udEx726[glr-1p::unc-7 + gcy-28dp::unc-7 + elt-2::gfp]*	This study
FG1171	*unc-7(e5); udEx727[glr-1p::unc-7 + gcy-28dp::unc-7 + elt-2::gfp]*	This study
FG1172	*unc-7(e5); udEx728[glr-1p::unc-7 + gcy-28dp::unc-7 + elt-2::gfp]*	This study
FG1173	*unc-7(e5); udEx729[glr-1p::unc-7 + gcy-28dp::unc-7 + elt-2::gfp]*	This study
FG845	*unc-9(e101); udEx491[unc-4p::unc-7 + elt-2::gfp]*	This study
FG846	*unc-9(e101); udEx492[unc-4p::unc-7 + elt-2::gfp]*	This study
FG847	*unc-9(e101); udEx493[unc-4p::unc-7 + elt-2::gfp]*	This study
FG1196	*unc-9(e101); udEx730 [unc-4p::unc-7 + odr-1p::unc-9 + elt-2::gfp]*	This study
FG1197	*unc-9(e101); udEx731 [unc-4p::unc-7 + odr-1p::unc-9 + elt-2::gfp]*	This study
FG1198	*unc-9(e101); udEx732 [unc-4p::unc-7 + odr-1p::unc-9 + elt-2::gfp]*	This study
FG1199	*unc-9(e101); udEx733 [unc-4p::unc-7 + odr-1p::unc-9 + elt-2::gfp]*	This study
FG842	*unc-9(e101); udEx488 [unc-4p::unc-7 + gcy-28dp::unc-9 + elt-2::gfp]*	This study
FG843	*unc-9(e101); udEx489 [unc-4p::unc-7 + gcy-28dp::unc-9 + elt-2::gfp]*	This study
FG844	*unc-9(e101); udEx490 [unc-4p::unc-7 + gcy-28dp::unc-9 + elt-2::gfp]*	This study

FG1226	*unc-9(e101); udEx734 [unc-4p::unc-7 + srbc-66p::unc-9 + elt-2::gfp]*	This study
FG1227	*unc-9(e101); udEx735 [unc-4p::unc-7 + srbc-66p::unc-9 + elt-2::gfp]*	This study
FG1228	*unc-9(e101); udEx736 [unc-4p::unc-7 + srbc-66p::unc-9 + elt-2::gfp]*	This study
FG1231	*unc-9(e101); udEx737 [unc-4p::unc-7 + trx-1p::unc-9 + elt-2::gfp]*	This study
FG1232	*unc-9(e101); udEx738 [unc-4p::unc-7 + trx-1p::unc-9 + elt-2::gfp]*	This study
FG1233	*unc-9(e101); udEx739 [unc-4p::unc-7 + trx-1p::unc-9 + elt-2::gfp]*	This study
FG1234	*unc-9(e101); udEx740 [unc-4p::unc-7 + trx-1p::unc-9 + elt-2::gfp]*	This study

Plasmids Generated for this Study

pFG383 *odr-1p::inx-7* cDNA

pFG384 *gcy-28dp::inx-7* cDNA

pFG385 *nlp-56p::inx-7* cDNA

pFG376 *odr-1p::inx-15* cDNA

pFG377 *gcy-28dp::inx-15* cDNA

pFG346 *odr-1p::inx-16* cDNA

pFG347 *gcy-28dp::inx-16* cDNA

pFG372 *gpa-4p::inx-16* cDNA

pFG373 *ceh-36p3p::inx-16* cDNA

pFG374 *str-1p::inx-16* cDNA

pFG343 *odr-1p::inx-17a* cDNA

pFG344 *odr-1p::inx-17b* cDNA

pFG336 *gcy-28dp::inx-17a* cDNA

pFG345 *gcy-28dp::inx-17b* cDNA

pFG348 *str-1p::inx-17a* cDNA

pFG349 *str-1p::inx-17b* cDNA

pFG350 *ceh-36p3p::inx-17a* cDNA

pFG351 *ceh-36p3p::inx-17b* cDNA

pFG352 *gpa-4p::inx-17a* cDNA

pFG353 *gpa-4p::inx-17b* cDNA

pFG375 *odr-1p::unc-7b* cDNA

pFG300 *gcy-28dp::unc-7b* cDNA

pFG399 *odr-1p::unc-9* cDNA

pFG298 *gcy-28dp::unc-9* cDNA

pFG387 *trx-1p::unc-9* cDNA

pFG386 *srbc-66p::unc-9* cDNA

Figure S1. ASH Modulatory Network

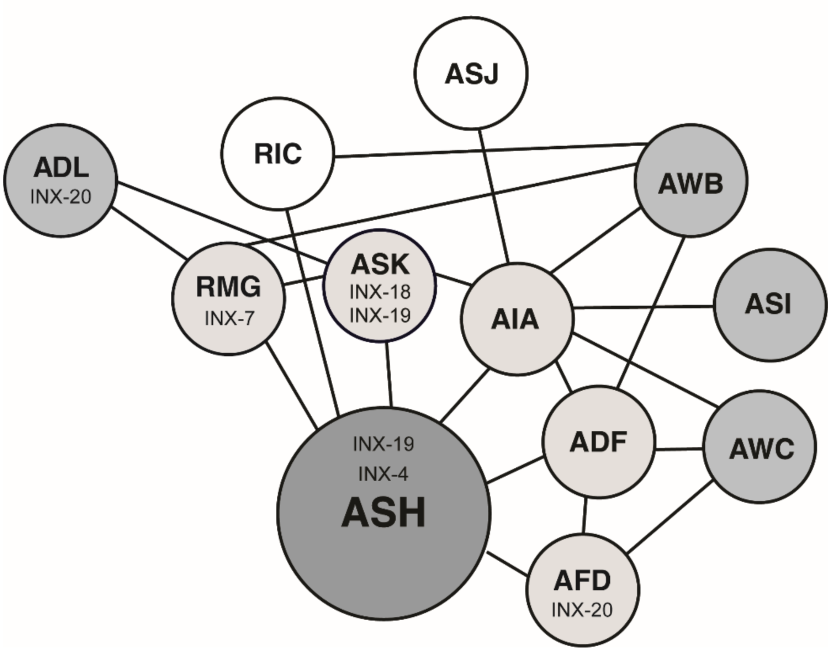

Supplemental Figure Legend:

Figure S1. ASH Modulatory Network

The sites of action of previously characterized innexins (INX-4, INX-7, INX-18, INX-19, and INX-20) are shown. The guanylyl cyclase ODR-1 functions to produce cGMP in AWB, AWC and AWC, while an unknown cyclase likely functions in ADL. Lines indicate anatomically defined gap junction connections based on the re-annotated neuronal wiring diagram (WormWiring.org) through which cGMP may pass to ASH [2,3]. Medium grey indicates neurons that produce cGMP. Light grey indicates neurons through which cGMP may pass. Figure adapted from [4].

Supplemental References:

1. Krzyzanowski, M.C.; Woldemariam, S.; Wood, J.F.; Chaubey, A.H.; Brueggemann, C.; Bowitch, A.; Bethke, M.; L'Etoile, N.D.; Ferkey, D.M. Aversive Behavior in the Nematode *C. elegans* is Modulated by cGMP and a Neuronal Gap Junction Network. *PLoS Genet* **2016**, *12*, e1006153, doi:10.1371/journal.pgen.1006153.

2. White, J.G.; Southgate, E.; Thomson, J.N.; Brenner, S. The structure of the nervous system of the nematode *Caenorhabditis elegans*. *Philos Trans R Soc Lond B Biol Sci* **1986**, *314*, 1-340, doi:10.1098/rstb.1986.0056.

3. Cook, S.J.; Jarrell, T.A.; Brittin, C.A.; Wang, Y.; Bloniarz, A.E.; Yakovlev, M.A.; Nguyen, K.C.Q.; Tang, L.T.; Bayer, E.A.; Duerr, J.S.; et al. Whole-animal connectomes of both *Caenorhabditis elegans* sexes. *Nature* **2019**, *571*, 63-71, doi:10.1038/s41586-019-1352-7.

4. Chaubey, A.H.; Sojka, S.E.; Onukwufor, J.O.; Ezak, M.J.; Vandermeulen, M.D.; Bowitch, A.; Vodickova, A.; Wojtovich, A.P.; Ferkey, D.M. The *Caenorhabditis elegans* innexin INX-20 regulates nociceptive behavioral sensitivity. *Genetics* **2023**, *223*, doi:10.1093/genetics/iyad017.
